# Developmental Differences in Children’s Learning and Use of Forensic Ground Rules During an Interview About an Experienced Event

**DOI:** 10.1037/dev0000756

**Published:** 2019-06-13

**Authors:** Deirdre A. Brown, Charlie N. Lewis, Michael E. Lamb, Jessie Gwynne, Oliver Kitto, Meghan Stairmand

**Affiliations:** 1 School of Psychology, Victoria University of Wellington; 2 Psychology Department, Lancaster University; 3 Department of Psychology, University of Cambridge; 4 School of Psychology, Victoria University of Wellington

**Keywords:** children, eyewitness testimony, ground rules, intellectual disability, interviewing

## Abstract

Children often answer questions when they do not have the requisite knowledge or when they do not understand them. We examined whether *ground rules* instruction—to say “I don’t know,” to tell the truth, and to correct the interviewer when necessary—assisted children in applying those rules during an interview about a past event and whether doing so was associated with more accurate accounts. We compared children with intellectual disabilities (mild or moderate severity, *n* = 44, 7–12 years) with 3 groups of typically developing children (2 matched for mental age, and 1 for chronological age, *n* = 55, 4–12 years) on their understanding of 3 ground rules, their use of these rules in an interview, and their accuracy in recalling a personally experienced event. Many children were able to demonstrate proficiency with the rules following simple instruction but others required additional teaching. Children applied the rules sparingly in the interview. Their scores on the practice trials of each rule were unrelated to each other, and to the use of the rules in context. Their developmental level was significantly related to both of these skills. Regression models showed that developmental level was the best predictor of children’s accuracy when they recounted their experience during the interview but that use of responses consistent with the rules, in conjunction with developmental level, predicted accurate resistance to suggestive questions. Future research should identify how best to prepare children of different ages and cognitive abilities to answer adults’ questions appropriately.

There are many contexts in which children are questioned by adults about their knowledge, experiences, and perspectives. The conclusions drawn from these interactions may have significant and far-reaching consequences. Researchers who question children interpret their responses in ways that may inform theory, practice, and policy. Clinical and educational psychologists score children’s responses on standardized tests and draw conclusions about their ability. Health professionals talking to children about their physical or emotional well-being develop diagnoses and treatment plans based on the children’s responses. Teachers make decisions about interventions and class placements based on assessments of the children’s learning. Social workers and police officers make decisions about children’s welfare based on children’s responses to forensic interviewers, whereas lawyers, judges, and jury members may subsequently make important decisions about others’ behavior based on the same responses. How, then, can we ensure that children are not unduly influenced by expectations about how they ought to answer adults’ questions (Lamb & Brown, 2006)? To ensure that we gain better information when making decisions about children’s well-being, we must prepare children to answer those questions appropriately (Malloy & Stolzenberg, 2018).

Scholars pointed out over 40 years ago (e.g., Donaldson, 1978) that children may interpret adult prompts in ways not expected by the questioners. For example, children may change their answers in cognitive tests investigating conservation if questions are asked again after the children did not respond correctly the first time (Rose & Blank, 1974). Similarly, children may answer nonsensical questions (‘Is red heavier than yellow?’) simply to please adult interrogators, despite recognizing the questions as nonsensical (Hughes & Grieve, 1980; Waterman, Blades, & Spencer, 2000). Children tend not to recognize that “I don’t know” is a valued response in a forensic setting (Scoboria & Fisico, 2013), given the encouragement (or even pressure) in other settings to answer questions even with guesses (e.g., in conversations with parents, or in tests of knowledge by teachers).

The social cultural theory of autobiographical memory highlights how early conversational interactions with significant adults pivotally shape how children talk about their past (Nelson & Fivush, 2004). Children are socialized from a young age to answer adults’ questions, so they may respond (convincingly) in the absence of sufficient comprehension and knowledge (Lamb & Brown, 2006; Lamb, Brown, Hershkowitz, Orbach, & Esplin, 2018). Much research has demonstrated how vulnerable children are to overt or implicit pressure from adults to respond to questions in a particular way (Klemfuss & Olaguez, 2018). Because children tend to comply with adults’ requests for information, researchers, interviewers, and clinicians typically instruct children how they should respond when, for example they are unsure. However, we know surprisingly little about how well these instructions prevent guessing or acquiescence and help children to challenge inaccurate statements or assumptions.

In many research protocols, as well as in other contexts where children are questioned about their experience (notably by health or forensic professionals), children are presented with *ground rules* at the outset of the conversation. These may include instructions to say “I don’t know” rather than guess, or to indicate whether they do not understand a question. Ground rule instructions are thought to alleviate some of the social challenges that children face when responding to adults’ questions (Brubacher, Poole, & Dickinson, 2015; Lamb & Brown, 2006; Malloy & Stolzenberg, 2018). From an early age, preschoolers learn how to recount their experiences (Neisser & Fivush, 1994), guided in large part by interactions with their parents (Nelson & Fivush, 2004; Salmon & Reese, 2015). Outside formal interviews, good stories may be more valued than accurate ones. For example, Kulkofsky, Wang, and Ceci (2008) showed that children who told better stories about events, from a narrative perspective, were often less accurate about the specific details than children who provided briefer accounts.

Ground rules are typically introduced with a brief instruction, perhaps accompanied by a very simple practice or demonstration example, which may have little relevance to the context to which it will be applied. However, it is doubtful whether such training to adopt a contrary response style overcomes socialized responding (Overton, 2010).

Because ground rules can be conceived as skills that children must acquire and apply to a new context, theories about transfer of learning and training might inform our expectations of how children should perform during an interview that follows instruction and practice using such ground rules. Scholars researching transfer of learning disagree about the extent to which we might expect learning of one new skill (e.g., responding “I don’t know” rather than guessing) to transfer to similar skills (e.g., saying “I don’t understand”) and to new problems (e.g., talking about a past event; Barnett & Ceci, 2002). Implicit in the notion of training is the expectation that children will both extract the relevant concept from the practice exemplars *and* recognize when to apply them in the subsequent interview, but it is unknown whether ground rules foment such metacognitive activity. Evaluating whether generalization actually occurs is therefore critical to evaluating whether ground rules are (a) understood by children and (b) assist them in managing their interactions with adults.

Research examining children’s learning in domains such as problem solving, language, categories, and numeracy has shown that the degree of similarity between practice examples and test problems affects how well children transfer learning to new domains (Day & Goldstone, 2012). Cognitive Load Theory (Sweller, Van Merrienboer, & Paas, 1998) and Progressive Alignment Theory (Gentner, Loewenstein, & Hung, 2007) both emphasize the importance of extensive rehearsal when teaching new concepts. Ground rules are seldom taught with multiple opportunities to practice the rules, however, and children’s understanding of how the rules may generalize to other kinds of questions is seldom assessed. Thus, the typical approach to the communication of ground rules is not consistent with psychological theories about how learning is promoted. We do not know how well these theories apply to new conversational rules. In the present study, accordingly, we sought to determine whether children understand and use ground rules when they are interviewed about a recent past event. In this way, we established a context analogous to one in which their behavior can have serious ramifications—forensic interviews with children about alleged maltreatment—but the results have broader relevance to any context in which children’s responses to adults’ questions are used to inform theory, policy, and decisions about children’s well-being (Malloy & Stolzenberg, 2018).

The conversational ground rules included in forensic protocols may also instruct children to provide unrestricted accounts of their experiences (e.g., “tell me everything, even the little things”), to report only what they are confident about (e.g., “don’t guess, just tell me what you really know”), and a statement about the interviewers’ naivety about what occurred (e.g., “I don’t know what happened”). Specifically, the most commonly included rules (Anderson, 2013; Brubacher et al., 2015; Lamb et al., 2018; Ministry of Justice, 2011), instruct children to tell the truth (e.g., “tell me the truth today, only tell me what really happened”), to say when they do not know the answer to a question (e.g., “if you don’t know the answer to a question, just say “I don’t know”), to signal if they do not understand a question (e.g., “If I say something that you don’t understand, you can just tell me”), and to correct the interviewer if they say something wrong (e.g., “If I make a mistake you should correct me”). However, there has been relatively little research on the extent to which children at different developmental levels make use of these instructions when recounting their experiences. Brubacher et al. (2015) recently conducted a study space analysis of the ways in which various ground rules have been evaluated. They concluded that there was only spotty evidence for the effectiveness of the various ground rules commonly included in forensic interviewing protocols. Even the best studied rule, the acceptability of saying “I don’t know,” has not been examined in conjunction with a number of factors known to influence *both* use of the rule (e.g., practice with the rule: Danby, Brubacher, Sharman, & Powell, 2015; Dickinson, Brubacher, & Poole, 2015) *and* children’s testimony (e.g., the effects of delay: Baker-Ward, Gordon, Ornstein, Larus, & Clubb, 1993; Jones & Pipe, 2002).

Only one study has examined children’s retention and application of these rules during interviews about personally experienced events (Danby et al., 2015). Danby et al. examined whether practice with a rule was associated with its spontaneous use during the interview and in response to three challenge questions at its conclusion (these questions were not related to the event children had experienced). They also examined whether children who practiced the rules were more accurate during the interview than children who had no practice. Danby et al. found that practice with the “don’t know” rule increased spontaneous use of it during an interview (compared to children who were instructed without practice), but practice with the rules did not lead to higher accuracy. Older children were less likely to say “I don’t know” than younger ones. Use of “I don’t understand” and the “correct the interviewer” rule was rare for all children and not affected by practice. They did not, however, examine whether children’s use of the rules in the interview was directly associated with accuracy of the information they reported.

Having established whether children can apply the rules, it is important also to determine whether doing so has any discernible impact on the quality of children’s accounts. To enact ground rules properly, and benefit from doing so, children must recognize when a rule ought to be applied (Barnett & Ceci, 2002). Brubacher et al. (2015) speculated about a range of cognitive processes that might be necessary for children to do this successfully. For example, they suggested that, to follow the rule of correcting interviewers if they get something wrong, children need to recognize that others can have false beliefs (Templeton & Wilcox, 2000). Some of the prerequisites identified by Brubacher and colleagues involve metacognition (e.g., monitoring one’s own knowledge state), perspective-taking abilities, and executive functions (e.g., working memory, inhibitory control). Children’s proficiency with many of these abilities is still developing well into middle childhood, and may vary according to task demands (e.g., Ceci, Fitneva, & Williams, 2010), raising questions about the developmental appropriateness of communicating ground rules to younger children and those with delayed or atypical development. Brown (1989) also argued that children need to understand or know the context to which they should transfer or apply new learning. Children taking part in a forensic interview, where the dynamics are so different from typical family and classroom interactions with adults (Lamb & Brown, 2006), may have difficulty effectively applying the ground rules even if they understand the embedded concepts.

We also know little about the extent to which performance on one type of ground rule might facilitate understanding or use of other ground rules. Cross-task correlations offer insight into shared underlying capacity (Barnett & Ceci, 2002), which has implications for the transfer of learning. Ground rules have typically been considered collectively, but proficiency with different rules may emerge at different developmental stages. Further, some scholars have suggested that transfer of learning may function differently depending on whether children are taught specific facts and procedures as opposed to general principles (Barnett & Ceci, 2002). In contemporary protocols, each ground rule represents a specific example of a broader conceptual category of responding (e.g., “don’t know” is a specific way of indicating uncertainty, “I don’t understand” is a specific way of indicating poor comprehension). As such, the rules may be too specific and situationally bound for appropriate transfer to the task of narrating a past event, especially given the limited surface and conceptual similarity between the two contexts.

Interviewing protocols and guidelines vary with respect to whether, which, and how ground rules are included and presented to children. When such rules are a formal part of the protocol, a single form of each ground rule is typically presented without accommodation for the age, cognitive- or information-processing ability, or developmental status of the children concerned (e.g., Lamb et al., 2018; La Rooy et al., 2015; Ministry of Justice, 2011). As Brubacher et al. (2015) observed, it is unlikely that children of different developmental levels are equally able to understand and apply these rules. Indeed, there is emerging evidence that children’s understanding (Dickinson et al., 2015), use of (Danby et al., 2015), and benefit from (Earhart, Rooy, Brubacher, & Lamb, 2014; Teoh & Lamb, 2013) such instruction varies with development.

An important omission from the literature on children’s understanding and use of ground rules when talking about the past is an examination of children with intellectual disabilities (CWID). This is not surprising, given their general underrepresentation in eyewitness testimony research and in legal proceedings (Brown, Lewis, & Lamb, 2015; Henry, Bettenay, & Carney, 2011), but their inclusion in studies of ground rules promises to improve our understanding of the developmental competencies needed to engage with such rules. CWID are at greater risk of maltreatment than typically developing (TD) children, and so understanding their particular needs when they are forensically interviewed may assist interviewers to interact effectively with them and increase their access to investigative and legal proceedings. Chronological age is the most robust predictor of performance across a number of dimensions relevant to children’s eyewitness testimony (Brown & Lamb, 2018; Klemfuss & Olaguez, 2018). It likely acts as a proxy for the dramatic increases that occur throughout childhood in cognitive (e.g., metamemory and strategy development, DeMarie, Miller, Ferron, & Cunningham, 2004, increases in knowledge and event representation, e.g., Brainerd & Reyna, 2012) and social understanding (e.g., learning how to talk about the past, Klemfuss, Rush, & Quas, 2016, conformity, acquiescence, e.g., Gudjonsson, Vagni, Maiorano, & Pajardi, 2016; Paz-Alonso & Goodman, 2016), in conjunction with neuromaturational development (e.g., prefrontal cortex, Ceci et al., 2010). Including children with intellectual disabilities in our research allows us to take advantage of the opportunity to distinguish between the independent effects of chronological and mental age by comparing children with typical and atypical developmental trajectories. For example, we can ask “are older children with a younger mental age comparable to typically developing younger children in their use of rules?”

CWID are likely to face several challenges in understanding, retaining, and applying ground rules when talking about past experiences and yet, paradoxically, are more likely to need this guidance. For example, language delays are common in CWID (Field, Allen, & Lewis, 2016; Pinborough-Zimmerman et al., 2007), meaning that these children may be more likely to (a) find questions incomprehensible and (b) have difficulty understanding the rules themselves (e.g., because the rules are lengthy and presented using grammatically complex sentences). CWID may also be more reliant than TD children on interviewers structuring the interaction because they are used to adults taking such roles during conversational exchanges (Hatton, 1998). CWID may thus not actively monitor their own comprehension of the questions, or assume that the adults will either fill in the gaps or reframe questions.

In this study, we extended previous research examining developmental differences in children’s understanding, retention, and application of ground rules in several ways. In one recent study, Dickinson et al. (2015) showed that children’s comprehension of different ground rules increased with age. Preschoolers (4- to 5-year-olds) had more difficulty with instructions to correct interviewers and to tell interviewers if they said something wrong. By about age 7, most children could demonstrate an accurate understanding of several ground rules in response to practice trials, and when questions were examined individually children generally did well. Across several ground rules, however, many (39%) of even the oldest children (8–9 years) failed at least one.

Danby et al. (2015) extended Dickinson et al.’s research by examining whether children, having demonstrated proficiency during a practice, subsequently made use of the rules during interviews or in response to some delayed test questions. We combined these two approaches, and examined children’s understanding of ground rule instruction at the beginning of interviews, their use of them during the interviews and when later suggestively questioned, and the association between both of these measures and the accuracy of the information children reported. Importantly, we examined TD children from a broader age range (4–12 years) than had Danby et al. (5–9 years) and included CWID in our sample in order to examine developmental changes associated with cognitive ability as well as both chronological and mental age variations in children’s performance.

We focused on three rules that are presented and practiced as part of the National Institute of Child Health and Human Development (NICHD) Investigative Interview Protocol: tell the truth, say “I don’t know,” and correct the interviewer. The NICHD Protocol is recognized as an effective tool for supporting interviewers to follow best-practice recommendations when conducting forensic interviews (Lamb, Orbach, Hershkowitz, Esplin & Horowitz, 2007; Lamb et al., 2018; La Rooy et al., 2015). Nevertheless, interviewers may still ask children difficult questions (e.g., those that stretch the limits of children’s recall, or those that are inadvertently suggestive), and make mistakes that require correcting. We used the NICHD Protocol to ensure that children’s use of ground rules was assessed in a context as similar as possible to the forensic context.

We therefore examined the responses of five groups of children (7- to 12-year-old CWID of either Mild or Moderate severity and 4- to 12-year-old TD children matched for either chronological (CA) or mental (MA) age) to the ground rules instruction and practice questions during the preparation stage of an interview. We also explored the children’s use of these rules during an interview about a staged, personally experienced event, and when responding to a series of highly suggestive questions that followed the interviews. We considered whether indices of rule understanding and use predicted children’s accuracy when describing the staged event during the interview and in response to suggestive questions at the end of the interview (some 25 min later).

We addressed the following research questions: (a) Are there developmental differences in children’s ability to understand instructions about ground rules and their use within interviews about personally experienced events? Based on previous research we predicted that performance during training and use of the rules during the interview would both increase with developmental level. (b) How does the grasp of one ground rule relate to the understanding of other rules? No research has examined this issue, but to the extent that the rules capture a common underlying capacity for metacognition, we might expect associations. On the other hand, if the concepts embedded within the rules are functionally dissimilar, we might expect no association. (c) Are there relationships between how children perform during training and within interviews, on the one hand, and the accuracy of their accounts, including answers to misleading questions, on the other? We predicted that children who were better able to correctly respond during training would be more likely to apply the ground rules during the interviews, and that doing so would be associated with higher levels of accuracy.

## Method

The study received ethical approval from the Lancaster University Research Ethics Committee (project title “Facilitating Eyewitness Testimony in Children With Learning Disabilities”; research protocol numbers were not used at the time).

### Participants

Ninety-nine children between 4 and 11 years of age participated in the study. Characteristics of the sample are presented in Table 1. All parents consented to the children’s participation, and the children gave verbal assent before being interviewed. Children were recruited from both mainstream and special schools in the Lancashire, Cumbria, and Yorkshire districts. The numbers of children in each group were: 21 in the CWID-Moderate group; 23 in the CWID-Mild group; 15 in the MA-Matched for the CWID-Moderate (henceforth MA-Moderate) group; 17 in the MA-Matched for the CWID-Mild (henceforth MA-Mild); and 23 in the CA-Matched (henceforth CA) group. Power analysis of the relation between ground rule understanding, use, and accuracy in the different groups of participants was not possible because this was the first study of its type. The subsamples were small but similar to those employed in other studies involving CWID who are, by definition, relatively uncommon and harder to access (Bettenay, Ridley, Henry, & Crane, 2014).[Table-anchor tbl1]

Assessment of ID was based on estimated IQ scores derived using a short form of either the Wechsler Preschool and Primary Scale of Intelligence (WPPSI-III U.K.) or the Wechsler Intelligence Scale for Children (WISC–III U.K.). The third editions were the most recent versions of these tests available at the time of data collection.

Consistent with the *Diagnostic and Statistical Manual of Mental Disorders*, fourth edition, text revision (*DSM–IV–TR*; American Psychiatric Association, 2000), which was in use at the time of data collection, participants were assigned to the CWID-Moderate group if their estimated IQ scores were between 40 and 55 (*M* = 48.81, *SD* = 2.89, range 44 – 53) and to the CWID-Mild group if their scores were between 55 and 79 (*M* = 67.70, *SD* = 7.13, range = 56–76). All CWID were capable of basic verbal communication (minimal phrase-based speech), confirmed in consultation with their teachers. Children in the TD group had estimated IQ scores within the average range (see Table 1). One child whose estimated IQ was 84 was included; this child was matched with a CWID whose IQ score was 20 points lower.

TD children were matched as closely as possible with CWIDs on the basis of gender and either CA or MA. MA was determined from the tables provided in the Wechsler manuals. When estimates were not available from the Wechsler manuals because the children’s ages fell in the crossover band between the two instruments, and the severity of ID made the range of MA estimates provided by the WISC–III U.K. insufficient, MA was estimated using: IQ = (MA/CA) × 100.

A check was made to examine the success of the matching strategy. A univariate ANOVA showed a significant main effect of chronological age for group, *F*(4, 94) = 68.19, *p* < .001, η_p_^2^ = .74. Tukey-Kramer tests showed, as expected, that children in the MA-Moderate group were significantly younger than the MA- Mild children who were younger than those in the other groups (all *p*s < .001: See Table 1), who did not differ from each other. A univariate ANOVA showed a significant difference in the average mental age of children in the different groups, *F*(4, 94) = 68.62, *p* < .001, η_p_^2^ = .74. Tukey-Kramer tests showed that children in the CA group had higher mental ages than those in both the CWID-Mild and their Matched MA group, who had higher mental ages than the children in the CWID-Moderate and their Matched MA group (all *p* < .01). Thus, the pairing of the children in each CWID subgroup with those in its comparison group in terms of MA and the equivalence of the CA matches for the two levels of ID was successful.

### Interviewers

Three female psychologists conducted the interviews. All had received training in use of the NICHD Investigative Interview Protocol. Deirdre A. Brown conducted frequent fidelity checks for adherence to the Protocol. CWIDs were recruited, in the main, from specialist schools, and so interviewers were typically aware of whether children had an intellectual disability (although not of the severity of impairment). Whether children were in the MA or CA matching group was evident from their age and class groups.

### Procedure

#### Event

The event was conducted in a room at school. Children were allocated to different teams (typically 5–6 children per group), led by a research assistant, and participated in three activities about first aid and safety. In one, children viewed large posters depicting dangerous hazards and discussed how the hazard might be overcome. In a second activity, participants watched a video-clip that showed a boy having a minor accident. The video demonstrated step-by-step care of minor cuts and abrasions. Children were taught and then asked to demonstrate how to take care of a simple cut, and applied a novelty sticking plaster they had selected. In a third activity, children learned how to tie a triangular bandage and practiced on each other. During this activity, the event leader took a photo of the children with their group leader. Part-way through the event, a fourth research assistant interrupted and staged a brief argument about the equipment. After completing all three activities, the groups gathered together to listen to a summary of what they had learned and each child received a small gift (novelty pencils).

##### Cognitive assessments

The Picture Completion, Information, Vocabulary and Block Design subtests of either the WISC-IIIUK or the WPPSI-IIIUK were administered during the week following the event (range 3–7 days).

##### Interview

The interviews were conducted at school one week after the event by the same interviewer who administered the cognitive tests. Each interview began with presentation of the ground rules (see below) and then proceeded to a combined rapport building and narrative practice phase using open-ended questions exploring what the children had done prior to the interview.

##### Ground rules

Each of the ground rules was explained in turn using the scripted instructions from the NICHD Investigative Interview Protocol (Orbach et al., 2000; see http://nichdprotocol.com/). The three rules were to (a) only tell the truth, (b) say “I don’t know” when appropriate, and (c) correct the interviewer if she made a mistake (see Table 2). The explanation of each rule was accompanied by an example, and then the child was asked to practice using each rule. Any children who did not respond correctly with the appropriate rule were given a second practice using the rule. If children still failed to correctly apply the rule after two practices, the rule was repeated verbally, and the interviewer progressed to the next rule (or the beginning of the interview if it was the final rule). Children were also instructed to tell the interviewer if they did not understand a question but this rule was not practiced.[Table-anchor tbl2]

##### Interview about the staged event

Focus was shifted to the staged event using a series of progressively informative prompts to help orient the children to the event the interviewers wanted them to talk about. The interview progressed using the prompts and structure outlined in the NICHD Protocol. *Open invitations* (e.g., “tell me about that time”) were used to encourage children to provide as much detail as possible. Children were encouraged to report further details using a variety of different prompts. Information reported by the children was used to form *cued invitations* (e.g., “you mentioned you got to choose a plaster; tell me more about choosing the plaster”), and children were also asked *direct questions* (open ended “wh-”, e.g., “which plaster did you choose?”), and *option-posing* questions, if needed, to clarify unclear or contradictory information (e.g., “did you or your partner wear the bandage first?). Interviewers were trained to follow responses to directive or option-posing prompts with open prompts (e.g., “tell me more about that”). *Suggestive* prompts reflected interviewer error (e.g., introducing information the child had not provided).

##### Scripted suggestive prompts

After the children indicated that they could not recall anything further they were asked 16 suggestive (leading and misleading) questions. Questions also varied depending on whether they were *closed* (e.g., “Were you in the blue group?”) or *open*, requiring the children to generate the information (e.g., “What color was the group you were in?”). Finally, questions varied depending on whether they assessed *central* or *peripheral* details about the event. Children were asked one of 12 sets of questions; across the sets we counterbalanced whether each topic (central or peripheral) was probed with a leading or misleading and closed or open questions.

Every interview was transcribed verbatim from the digital video recordings. All interviewer and child utterances were included.

### Coding

#### Child responses

Two separate coding schemes were developed, one for the information reported during the NICHD Protocol interview, and one for responses to the suggestive questions. The lead rater was not blind to the group membership of each child (CWID vs. MA vs. CA); participants tended to be grouped by the school they attended and it was not possible to remove this detail from the transcripts. A subset (10%) of the interviews conducted was coded by a member of the research team (blind to the group membership of the child) to assess intercoder reliability and ensure that awareness of group membership had not affected how the interviews were coded, and the lead coder also recoded a different subset of the interviews (10%) to check for drift. The mean Cohen’s kappa value of .91 was high (Cohen, Cohen, West, & Aiken, 2003). Children’s accuracy during the interview was calculated by the proportion of correct pieces of information relative to the total amount of information provided. Accuracy of responses to the suggestive questions was calculated by dividing the number of correct responses by the total number of questions asked.

#### Ground rules

Two coders (blind to the hypotheses) coded the children’s responses to the ground rules. Children were given a score of 2 if they correctly responded to the practice item for a rule (for a maximum score of 6 across the three rules). One point was given if children required the second practice example, but then correctly responded to that item. A score of zero was given to children who did not demonstrate correct use of the rule after two practice trials. Thus, the range of possible scores totaled across the three rules was 0–6. Each coder coded every transcript, and differences were resolved by discussion. Reliability was again high with a mean kappa of .91.

A different research assistant (also blind to the study hypotheses and to the accuracy of children’s responses) coded the use of the ground rules during the interviews and the suggestive questioning. Children’s use of the explicit language or behavior targeted by each rule was scored, as were alternative behaviors that could be considered as implicit, rather than explicit, demonstrations of the rule. The range of responses coded, with associated definitions and examples, are presented in Table 3. A second assistant coded 40% of the transcripts for reliability. Reliability was high (Cohen’s κ = .88 during the interviews and .85 in response to suggestive questions).[Table-anchor tbl3]

## Results

### Are There Developmental Differences in Children’s Understanding of Ground Rules?

We started by asking whether the participants were capable of following three ground rules after brief instruction and a test question. Irrespective of group, the children responded well to the trials assessing understanding of each of the rules. To the first test questions about all three rules, 56% of the children responded correctly. Given this negative skew, we used ordinal regression to compare the two groups of ID children (ID-Mod and ID-Mild), the two MA-matched groups (MA-Mild and MA-Mod), with the CA group as the comparator. In all but one case, the test of parallel lines was nonsignificant, suggesting that the assumptions of this procedure had not been breached. In the exceptional case (focusing on the “tell the truth” rule) we used a linear chi-square test.

Table 4 presents the mean scores, summaries of the statistical analyses, and the individual group comparisons on the ground rules questions. It shows that all the tests revealed significant effects (see model statistics column). When responses to the three sets of questions were combined (TOTAL), the parameter estimates indicated that children in both of the CWID groups and the Moderate-MA comparison group were more likely to make errors than participants in the CA group. For the first rule (tell the truth), the chi-squared showed a linear effect: as ability group increased from CWID-Moderate through to CA so did the number of children correctly responding on the first trial. For the “don’t know” rule, children in the CWID-Moderate group and the Moderate-MA group made more errors than children in the CA group. For the “correct me” rule, children in the Moderate-MA group made more errors than children in the CA group (although two thirds of the children in this group were correct). Thus, in response to Research Question 1, and consistent with our hypothesis, with increased mental age children become better equipped to understand each ground rule question.[Table-anchor tbl4]

### Relationships Between Ground Rules Performance

We examined whether performance on each ground rule was related to the other two. There were no significant associations (in all cases, *r* < .08) between children’s success at responding to the practice trials of each rule. Concerning Research Question 2, grasping one rule appeared not to be related to understanding another, supporting the second of our proposed relationships. The bottom two rows of Table 5 report the children’s accuracy both when describing the experienced event when interviewed using the NICHD Protocol, and in response to the 16 scripted suggestive questions at the end of the interview. They show that, with some variations between groups, 85% of the children’s interview responses were correct but that accuracy dropped to 59% for responses to the suggestive questions.[Table-anchor tbl5]

### Understanding Rule Use, Using Rules, and Accuracy in the Interview

The rest of the analyses unpacked Research Question 3. Performance on each of the three ground rule questions was correlated with the accuracy of the children’s accounts, *r*(98, 96,^1^ and 98, respectively) = .23, .39, and .28, all *p* ≤ .02, for the three respective rules, and with the accuracy of the children’s responses to the suggestive questions at the end of the interview (respectively, *r*[98, 96, and 98, respectively] = .23, .25 and .25; all *p* ≤ .02). To reduce the variables, we collapsed the various response behaviors that were consistent with nonsubstantive responses to the interviewers’ questions (“don’t know,” “not sure,” “can’t remember,” and “don’t understand”), and corrective responses (corrects interviewer, and refutes interviewer), in part because many of the individual response categories were infrequently observed. Table 5 reports the findings for use by children in each group of these two overarching response categories in the main interview and in response to the suggestive questions. We next examined whether children’s nonsubstantive and corrective responses differed by Group and performance on each of the learning trials at the beginning of the interview. We conducted a series of repeated measures ANOVAs with response type (nonsubstantive vs. corrective) as the within-subjects factor and Group as the between-subjects factor. We then added performance on each rule as a continuous covariate. Given the positive skew for three of the four response categories included in Table 5, preliminary analyses confirmed that parametric analyses could be performed (see footnote for Table 5; raw means are presented).

In the first analysis on the effects of utterance type and group, there was a significant difference in response type, *F*(1, 94) = 319.7, *p* < .001, η_*p*_^2^ = .77: Children gave more nonsubstantive responses (*M* = 10.41, *SD* = 8.00) than corrective responses (*M* = 1.22, *SD* = 1.63). There was also a significant effect of Group, *F*(4, 94) = 3.45, *p* = .01, η_*p*_^2^ = .13: Tukey-Kramer tests showed that children in the CA group gave fewer responses (irrespective of type) than children in the MA-Moderate group (*p* < .05). When we added each of the measures of rule understanding, none made significant contributions to the model (Truth or lie: *F*[1, 93] = 0.04, *p* = .85, η_*p*_^2^ = .00; Do not know: *F*[1, 93] = 0.48, *p* = .49, η_*p*_^2^ = .01; Tell the truth: *F*[1, 93] = 0.00, *p* = .97, η_*p*_^2^ = .00), nor were any interactions significant. Thus, our hypothesis relating to understanding of the ground rules and their subsequent use was not supported.

A similar set of analyses exploring children’s use of nonsubstantive and corrective responses during the scripted suggestive questions phase used raw data because the distributions allowed this. When Response type and Group were entered into the model, there was a main effect of Response Type, *F*(1, 94) = 19.7, *p* < .001, η_*p*_^2^ = .17: Children made more corrective (*M* = 5.14, *SD* = 2.4) than nonsubstantive responses (*M* = 3.13, *SD* = 2.9). The effect of group was not significant, *F*(4, 94) = 2.21, *p* = .07, η_*p*_^2^ = .09, but the Group × Response Type interaction was *F*(4, 94) = 2.6, *p* = .04, η_*p*_^2^ = .1. To unpack this interaction, we conducted univariate analyses on each response type. For corrective responses, the main effect of Group was significant, *F*(4, 94) = 5.61, *p* < .001, η_*p*_^2^ = .19, and Tukey’s tests showed that children in the Moderate ID group gave fewer responses than those children in each of the other groups (Tukey *p*s < .05). For the nonsubstantive responses, the effect of Group was not significant, *F*(4, 94) = 0.75, *p* = .56, η_*p*_^2^ = .06. When we added each ground rule score separately, the main effect of Response Type and the Group × Response Type interaction remained significant. The Ground Rules “truth or lie” and “don’t know” did not add to the models (in both cases the main effects and interactions, *F*< 1), but the use of “correct me” × Response Type interaction did, *F*(1, 94) = 4.09, *p* = .03, η_*p*_^2^ = .04. Follow up analyses showed that the main effect of corrective utterances showed an effect of a grasp of the “Correct me” rule, *F*(1, 94) = 5.04, *p* = .02, η_*p*_^2^ = .11, but the children’s use of the nonsubstantive rule did not, *F*(1, 94) = 2.07, *p* = .15, η_*p*_^2^ = .02. Thus we found partial support for our hypothesis that ground rule performance would be associated with higher rates of rule related responding.

### Understanding and Use of the Ground Rules in the Interview and the Accuracy of Children’s Accounts

The three left-hand columns in Table 6 present the intercorrelations between the children’s use of nonsubstantive and corrective responses during the main interview and to the scripted suggestive questions. Production of nonsubstantive responses in the interview was positively correlated with the use of corrective responses during the same phase and with nonsubstantive responses when responding to the subsequent suggestive questions. Children’s corrective responses in the interview were also positively correlated with corrective responses to the suggestive questions. Children’s corrective responses to suggestive questions were negatively correlated with avoidant responses during this phase. The two right-hand columns of Table 6 show the correlations between the children’s use of nonsubstantive and corrective terms in the interview and their resistance to misleading questions in relation to the accuracy of their statements. Nonsubstantive responses during the interview were not correlated with the accuracy of children’s statements in this phase but were negative correlated with accuracy responding to the suggestive questions. Corrective responses during the main interview were negatively correlated with accuracy during this phase but not related to accuracy when responding to the suggestive questions. Nonsubstantive responses during the suggestive questions phase were not correlated with accuracy during the main interview but were negatively correlated with accuracy responding to the suggestive questions. Corrective responses to the suggestive questions were positively correlated with accuracy during both the main interview and the suggestive questions phase.[Table-anchor tbl6]

Finally, we conducted two sets of hierarchical multiple regressions to examine whether accuracy in the final two phases of the interview were predicted by the children’s use of the ground rules in the interview, their developmental level, and their knowledge of ground rules at the outset. The first step of each analysis examined whether the children’s nonsubstantive and corrective responses in both the main interview and separately in response to the suggestive questions predicted the accuracy of the information reported in the interview or in response to the suggestive questions (see Table 7). In each set of analyses, we checked for collinearity and, as the Variance Inflation Factor values were all less than 1.5, we left all the variables in the models.[Table-anchor tbl7]

In both regressions, the first step in the model was significant. Three of the response types in the main interview predicted accuracy in the interview itself: correcting the interviewer during the main interview negatively predicted accuracy in that phase while corrective and nonsubstantive responses in the suggestive questions phase were positively related to accuracy (see Step 1 of the first analysis in Table 7). In the second step, we added group (ordered by developmental level) and the scores on the three ground rule test questions during the preparation phase. The addition of these four additional variables explained significant variance to the model and changed it: the use of correctives in the main interview remained a negative predictor, while employing nonsubstantive responses in the same phase of the interview was positively related to accuracy. Group membership explained significant variance as did one of the three measures taken in in the presubstantive phase—the ability to respond to the “correct me” rule (Table 7, Step 2 in the first panel). Again, we found partial support for our hypothesis that performance during training and use of rules would be associated with higher accuracy, although the direction of the associations was not wholly consistent.

When we examined accuracy during the suggestive questions phase, three of the response types in the interview predicted accuracy: both correcting the interviewer in the main interview and nonsubstantive responses during the suggestive phase negatively predicted accuracy, while correcting the interviewer in the suggestive phase positively predicted accuracy. In the second step of the analysis, correcting the interviewer during the suggestive phase remained a significant positive predictor, nonsubstantive responses during that phase negatively predicted accuracy (but note that the raw correlation was nonsignificant in Table 6), and group membership was again a significant positive predictor. Here, then, there was not support for our hypothesis that training performance would be associated with use of rules, although there was an association between use of rule-related responses and accuracy (again with inconsistency with respect to the direction of the relationship).

## Discussion

The results raise four issues that we discuss in turn. First, they suggest developmental differences in children’s ability to understand ground rules during instruction, or to apply them either when describing experienced events, or when responding to highly suggestive questions. Second, successful performance on the different ground rule practice trials did not uniformly translate to more accurate responding when recalling a past experience, with the “Correct Me” rule being the only consistent predictor of accuracy during the main interview. Third, even though use of responses consistent with some of the ground rules predicted accuracy during the main interview when considered in isolation, developmental level was a stronger predictor of how well children recounted their experiences. However, when responding to suggestive questions, both the ground rules-consistent with responses to those questions and developmental level explained unique variance in the accuracy scores. Fourth, the fact that performance on the ground rules did not consistently predict accuracy during the interview and did not predict accuracy when responding to suggestive questions at all raises questions about the amount and type of instruction required to enhance children’s understanding of what is expected of them when they are being questioned.

There were clear developmental differences in each phase of the interview. The children’s ability to utilize the ground rules correctly during the preparation phase improved with developmental level, as did their use of rule-related behaviors during the interview and in response to scripted suggestive questions. Although many children passed all rules, as they did in previous research (Dickinson et al., 2015), a sizable minority (44%) failed at least one, and this was most common in the children with lowest mental ages (86% of the MA-Moderate group, and 80% of the CWID-Moderate group) and those with mild cognitive impairments (43%). In contrast to Dickinson et al.’s (2015) findings, the children with higher mental ages in our sample were almost always correct on all of the rules, whereas 40% of the oldest children in their study were below ceiling. This difference may reflect the ages of the children in the two studies. The oldest children in Dickinson et al.’s study were 9 years old (*M* = 6;5), whereas our sample included children up to 11 years old (*M* = 10;5). However, the MA of children in our MA-Mild group was 7 years, and 82% of these children responded correctly on the first trial of all of the rules. Perhaps, then, the differences also reflected the nature of the rule instruction and practice examples?

Previous studies have suggested that the “Don’t know” rule is the easiest for children to understand (e.g., Danby et al., 2015; Dickinson et al., 2015), but many children in our sample had some difficulty with this rule. Half of the CWID-Moderate children and about a third of the youngest TD children (MA-Moderate) failed on the first trial. However, most of the children in our sample showed more proficiency with the “correct me” rule than those in other studies.

Our study did not allow a deeper exploration of the mechanisms accounting for the developmental shifts observed. Simple conceptions anchored by chronological age were insufficient to account for the observed developmental differences. As noted by other researchers, younger children have multiple memory encoding and retrieval problems, in part because their prefrontal cortexes (PFC) are immature (Ceci et al., 2010). Neurological changes are important but not sufficient to account for developmental differences in metacognition and memory, however, so other developing abilities may play a greater role in explaining developmental changes in metacognitive tasks (Ceci et al., 2010). The older children with IDs, who may have experienced the necessary PFC development, were often most similar to younger children in our study, indicating that they lacked other important abilities. In fact, typically developing school-age children still have memory difficulties despite mature PFCs because they lack the ability, not only to link objects to all their attributes successfully, but to properly introspect about what they know and how they are being asked to use that knowledge. Ceci et al. (2010) have proposed that this difficulty reflects, in essence, a representational issue that contributes to metamemory. In terms of our results, the implication is that younger children (and presumably also children with IDs) might fail to apply ground rules not because they do not remember them or cannot respond to them in simple practice trials but because they cannot recognize the relevance of ground rules to specific situations in which they would be relevant. We need further research exploring the gap between remembering or responding to simple ground rule tests and applying them during interviews with a focus on understanding children’s ability to accurately identify the situations in which they should be applied. As Brubacher et al. (2015) suggested, we need to know more about children’s comprehension of the concepts that underlie the various rules, and the causes of some children’s poor understanding and use of them. Lessons from educational and cognitive psychology about conditions that facilitate transfer of training may contribute to developing this understanding (Barnett & Ceci, 2002; Day & Goldstone, 2012; Gentner et al., 2007; Sweller et al., 1998).

We saw developmental differences in rule-related responses during the interview. Accuracy can be considered a proxy for telling the truth, nonsubstantive responses are consistent with the instruction to say “I don’t know”, and corrective responses are consistent with the “correct me” rule (Danby et al., 2015; Earhart et al., 2014). As in Danby et al.’s (2015) study, children used corrective responses sparingly during the interview. However, our participants used more nonsubstantive responses than the children in their study, perhaps because we considered a wider range of behaviors as examples of nonsubstantive responding.

Theories of transfer of training suggest that children’s greater competency with particular rules at different ages may reflect the types of examples given during instruction and the questions used to test them (Dickinson et al., 2015). In our study, the test question for the “don’t know” rule (“What’s in my pocket?”) may have prompted a guess (e.g., “money”), thereby necessitating additional instruction and practice. In contrast, the “correct me” practice trial (“If I said you were a three-year old boy” to a 5-year-old girl) may have more easily elicited a correction. Thus, single practice questions are unlikely to capture the complex applicability of each rule. They also do not exemplify the narration of personal experiences and are thus unlikely to optimally prepare children to use ground rules during such interviews (Brown, 1989; Overton, 2010).

At least one source of difficulty impeding the application of ground rules during an interview is the disconnect between how children are trained to use them and the context in which the rules are meant to be applied (Barnett & Ceci, 2002). Although children below 7–8 years of age *can* benefit from instruction in memory strategies, they may fail to use such strategies spontaneously unless explicitly prompted to do so (Brown & Pipe, 2003a, 2003b). Even then, they may not benefit from use of the strategy (Bjorklund, Miller, Coyle, & Slawinski, 1997) or fail to generalize new learning to novel tasks (Borkowski, Milstead, & Hale, 1988). Research examining children’s learning in domains such as problem solving, language, categories, and numeracy, has shown that the degree of similarity between practice examples and test problems affects how well children transfer learning to new problems (Day & Goldstone, 2012), as does the number and nature of the practice items offered (Braithwaite & Goldstone, 2015; Sweller et al., 1998). Barnett and Ceci (2002, p 632) suggested that “general heuristics and principles may transfer more readily than more specific learning.” Perhaps, then, ground rule instruction would be more effective if children were taught the broader principle of “you don’t have to try to answer the questions” rather than specific exemplars of each rule or potential problem that may be encountered in the interview.

The second main finding concerns the lack of association between children’s adherence to each of the rules during the instruction and practice. We saw no evidence of a shared capacity for metacognition. Even taking the differences between individual examples into account, the various ground rules differ conceptually and require different skills and understanding. For example, only making truthful statements is very different from admitting ignorance or correcting an adult’s error. Our data suggest that future research should examine ground rules in relation to the separate and unique skills that contribute to the reliability of children’s responses to questions (Donaldson, 1978), and we should be wary of assuming that the application of ground rules, at least as they are currently conceptualized and taught, involves a single skill. However, the children did use two of the rules consistently in the interview and suggestive questions phases. In conjunction with the developmental differences observed in the understanding and use of the various rules, it appears that a “one size fits all” approach to selecting and presenting the different rules is unlikely to create an optimal context for children of different developmental levels. Instead, as suggested above, a broader conceptual approach rather than a focus on specific forms of responding may be more useful.

The third finding derived from the regression analyses. Despite the presumption that rehearsing ground rules improves the quality of children’s recall, protecting against the adverse impact of risky questions, performance on the rules was not universally associated with accuracy in the interview; developmental level explained more variance. Only the “correct me” rule contributed to accuracy once the effect of group was taken into account. As Brubacher et al. (2015) pointed out, the various ground rules have not all been examined to the same extent. There is evidence that encouraging children to say “I don’t know” as needed (alongside encouragement to respond when children do know the answer) can enhance accuracy (Gee, Gregory, & Pipe, 1999; Saywitz & Moan-Hardie, 1994), although the relevant research largely examined responses to misleading questions. Within this study, frequent requests to elaborate upon their previous comments reduced the number of questions requiring “don’t know” responses. However, even when field interviewers follow a best practice protocol, they may still ask some problematic (complex or suggestive) questions (Brubacher et al., 2015; Cyr & Lamb, 2009; Danby et al., 2015; Dickinson et al., 2015; Earhart et al., 2014; Lamb et al., 2009; Orbach et al., 2000). We do not know enough about the extent to which such questions affect reliability as much as when a set of highly leading or misleading questions are administered in a block (Brown & Lamb, 2018). Brown et al. (2013) showed that the accuracy of children’s responses to suggestive questions within an NICHD Protocol interview were no lower than those given to more appropriate questions. Thus, it is unclear whether ground rule instruction improves the quality of children’s responses to mildly suggestive questions nested within an otherwise appropriate interview.

Fourth, the links between the children’s use of ground rules and accurate responding were complex but suggest some clear guidance for practitioners and researchers. Answering suggestive questions with nonsubstantive responses negatively predicted children’s accuracy, whereas correcting the interviewer positively predicted it, even when developmental level was considered, overriding any effects of earlier ground rule knowledge (see Table 7). Corrective responses particularly protected against the effects of suggestive questioning (where accuracy dropped to 59% compared to 85% when children were interviewed appropriately). However, fewer corrective responses were associated with greater accuracy during the main interview. Thus, preparation to correct may be especially important when children are cross-examined, often using the types of questions posed in the suggestive phase (Andrews & Lamb, 2017; Zajac, O’Neill, & Hayne, 2012). Indeed, practice and feedback about how to manage such questions can reduce inconsistency (Righarts, O’Neill, & Zajac, 2013). In contrast, preparation to indicate uncertainty (nonsubstantive responses) may be especially important for interviews that elicit children’s own accounts using strong interview protocols.

By identifying varying competencies, we could better advise researchers and practitioners on how best to select fit-for-purpose tools that are tailored to the children being interviewed (e.g., Saywitz & Camparo, 2014). Offering more comprehensive instruction about appropriate ground rule usage can also occur while developing rapport and assisting children to settle into their unique roles as knowledgeable informants (e.g., Brown et al., 2013; Roberts, Brubacher, Powell, & Price, 2011).

### Limitations

This study examined recall of an experienced event, and so our analyses were limited to observing whether children naturalistically demonstrated rule use when interviewed. The relatively low incidence of rule-related strategies may reflect the predominantly open-ended interview style and the limited instances where children might have needed to use the rules, as indicated by the higher incidence of corrective responses during the 16 scripted suggestive questions. Future research should examine adherence to the ground rules when children are asked questions that should elicit such responding, particularly when such questions are also embedded within ecologically valid interviewing paradigms.

More than half of the children in our study could follow each rule after minimal training. We were unable to determine whether this reflected successful training or preexisting mastery, and therefore whether children who understood how to apply the rules differed from those who learned this during instruction. Including a no-instruction control condition would be useful in considering this question. We also acknowledge that the event about which the children were interviewed was positive and socially sanctioned, which may mean children interacted differently during the interview than they might while being interviewed about possible maltreatment. Finally, given the small numbers within each developmental and cognitive group in our sample, and the variance in findings across studies to date, replication is important.

### Implications and Future Directions

Question format can profoundly influence the nature of both TD and CWID’s responding in several tasks and settings (e.g., Brown et al., 2013; Brown, Lewis, Lamb, & Stephens, 2012; Lamb et al., 2018; Waterman et al., 2000). As discussed earlier, important directions for future research concern how to present the rules, demonstrate the ways in which they should be used, and facilitate the transfer of learning, especially by younger and developmentally delayed children. For example, visual cues for important types of information can enhance reporting by children trained to use them (e.g., Saywitz & Camparo, 2014). Whether reminders of the ground rules in later stages of an interview (which are a feature of some protocols) facilitate children’s use of them also needs to be examined.

Our results demonstrate that instruction in various rules and children’s implicit and explicit applications of these rules may affect the reliability of their reports, Importantly, the degree to which children understand and apply the various rules changes as development progresses. Eliciting information from children is a key aspect of developmental science and is also critical in a range of applied contexts. When health professionals ask children about their symptoms, accurate answers are crucial for diagnosis and effective treatment (Waterman & Blades, 2011). When researchers use interviewing methods to assess knowledge, their conclusions will be influenced by the children’s responses (Fritzley & Lee, 2003). In police officers’ or social workers’ interviews about possible maltreatment, the veracity of children’s testimony determines whether charges will be laid (Brown & Lamb, 2015). Developing a better understanding of how children learn and use conversational rules during interactions with adults is important, not only within these applied settings, but also for establishing the optimal conditions in which children can demonstrate their understanding.

## Figures and Tables

**Table 1 tbl1:** Characteristics of the Sample

Characteristic	CWID (moderate)	CWID (mild)	MA matched (moderate)	MA matched (mild)	CA matched
*N*	21	23	15	17	23
*N* (male)	17	15	9	9	9
*N* (female)	4	8	7	8	14
*M* age in months (*SD*)	118.67 (13.35)	115.96 (14.83)	62.4 (8.31)	84.71 (11.73)	123.52 (15.41)
*M* mental age in months (*SD*)	62.71 (9.85)	83.17 (11.31)	64.67 (12.62)	87.12 (12.39)	133.57 (25.67)
*M* estimated IQ (*SD*)	48.81 (2.89)	67.70 (7.13)	101.0 (11.03)	101.00 (10.8)	104.96 (11.08)
Range of estimated IQ scores	44–53	56–76	88–118	87–118	84–124

**Table 2 tbl2:** Ground Rule Instructions and Practice Trial Questions

Ground rule	Instruction	First practice trial	Second practice trial
Tell the truth	When we talk today you should only tell me about things that are really true, that really happened to you.	If I said that you took your shoes off when you came into this room, would that be true or not true?	If I said that you were wearing a green shirt now [when child is wearing a different color], would that be true or not true?
Say “I don’t know”	When you don’t know, you don’t have to guess, its okay to say “I don’t know”.	If I ask you what’s in my pocket, what would you say?	If I asked you what is in my bag over there, what would you say?
“Correct me”	If I say things that are wrong, you should correct me	If I said that you were a 3-year-old girl (when interviewing a 5-year-old boy etc.) what would you say?	If I said that you were standing up, what would you say?

**Table 3 tbl3:** Coding of the Ground Rules Test Questions

Type of response	Response	Definition	Example
Nonsubstantive responses	Don’t know	The child communicates (either verbally or non-verbally) that they don’t know information	C: “Sam S was working with Jack [I nods], I don’t know anymore though about who the other people were working with”
	Can’t remember	The child communicates (verbally) that they can’t remember information	C: “Well I can’t remember any more about how you put on the slings”
	Not sure	The child communicates (verbally) that they’re not sure	I: “A lady came in during the safety activities. What hospital did she have to go to?”
			C: “I’m not sure, maybe Lancaster, but I’m not sure about that [shakes head].”
	Don’t understand	The child communicates (verbally) that they don’t understand the interviewer’s question	I: “How did you know you were in the orange team?”
			C: “[pause] because. . .I don’t get that, I don’t get that question.”
Corrective responses	Corrected	The child corrects (verbally or non-verbally) the interviewer’s interpretation	I: “The girl on the video hurt her arm. Which part of her arm did she hurt?”
			C: “It was a boy.”
	Refuted	The child refutes (verbally or non-verbally) the interviewer’s interpretation	I: “Okay. What color was the hat you put on?”
			C: “I didn’t put a hat on”

**Table 4 tbl4:** Mean (SD) Scores for Test Questions for Each Ground Rule in the Preparation Phase by Group (Range 0–2, 0–6 for Total) and Percentages of Children Passing Each Rule on the First Trial

Rule	CWID-Moderate (*n* = 21)	MA-Moderate match (*n* = 15)	CWID-Mild (*n* = 23)	MA-Mild (*n* = 17)	CA match (*n* = 23)	Model statistics χ^2^(*df* = 4)	Wald tests χ^2^(*df* = 1)	Comparisons with CA group
Tell the Truth	1.62 (0.67)	1.4774 (0.5244)	1.70 (0.47)	1.88 (0.49)	2.0 (0.00)	11.40,^a^ *p* = .001, Cramer’s V = .44		MA < CA
% children correct on first trial	71	47	70	94	100			
Don’t know	1.30 (0.80)	1.570 (0.7660)	1.78 (0.42)	1.88 (0.33)	1.96 (0.21)	17.27, *p* = .002, *R*^2^ = .22	All > 5.0, all *p* ≤ .02	CWID-Moderate + MA-Moderate < CA
% children correct on first trial	50	64	78	88	96			
Correct me	1.71 (0.56)	1.53 (0.74)	1.96 (0.21)	2.0 (0.00)	1.96 (0.21)	14.93, *p* = .005, *R*^2^ = .24	4.56, *p* = .03	CWID-Moderate < CA
% children correct on first trial	76	67	96	100	96			
Total	4.60 (1.0)	4.50 (1.16)	5.43 (0.73)	5.76 (0.56)	5.91 (0.29)	44.06, *p* < .0001, *R*^2^ = .41	All > 6.2, all *p* ≤ .01	CWID-Moderate + CWID-Mild + MA-Moderate < CA
^a^ χ^2^(*df* = 1).								

**Table 5 tbl5:** Mean (SD) Scores for the Use of Nonsubstantive and Corrective Ground Rule Responses by Children in Each Group During the Interview and in Response to Suggestive Questions, and the Accuracy of Responses in the Interview and in Response to Suggestive Questions

Type of response	CWID-Moderate	MA-Moderate	CWID-Mild	MA-Mild	CA match
Interview					
Nonsubstantive responses^a^	11.09 (9.07)	14.07 (8.99)	11.96 (8.7)	10.76 (8.93)	7.43 (4.5)
Corrective responses^a^	1.43 (1.69)	2.20 (2.24)	1.48 (1.73)	1.0 (1.22)	.30 (.56)
Suggestive questions					
Nonsubstantive responses^a^	3.76 (3.49)	2.53 (2.5)	3.82 (3.7)	3.53 (2.64)	2.74 (2.36)
Corrective responses	3.24 (2.55)	6.27 (2.58)	5.78 (2.56)	5.35 (1.87)	5.78 (1.98)
Accuracy					
Interview	0.77 (0.10)	0.82 (0.07)	0.86 (0.07)	0.90 (0.04)	0.91 (0.04)
Suggestive questions	0.39 (0.14)	0.57 (0.22)	0.62 (0.21)	0.63 (0.12)	0.72 (0.15)
^a^ Variable log transformed and outliers adjusted.					

**Table 6 tbl6:** Correlations Between Children’s Use of Ground Rules in the Interview or in Response to Suggestive Questions and the Accuracy of the Children in the Interview and Suggestive Questions Phases (Two Right-Hand Columns)

Type of response	Corrective in interview^a^	Nonsubstantive in suggestive^a^	Corrective in suggestive	Accuracy: Interview	Accuracy: Suggestion
Nonsubstantive in interview^a^	.23*	.5**	−.00	−.03	−.19
Corrective in interview^a^		.05	.24*	−.26**	−.09
Nonsubstantive in suggestive^a^			−.35**	.08	−.45***
Corrective in suggestive				.23*	.60***
^a^ Variable log transformed and outliers adjusted.					
* *p* < .05. ** *p* < .01. *** *p* < .001.					

**Table 7 tbl7:** Results of Hierarchical Regression Exploring the Impact of the Use of Trained Terms in the Interview (Step 1) and Group and the Children’s Success at the Three Preparation Training Trials (Step 2) on Children’s Accuracy During the Interview and Scripted Suggestive Questions

Model	Step	Predictor variables	*b*	SE	β	*t*	*p*	Adjusted *R*^2^
Accuracy in main interview	1	Nonsubstantive: main	.01	.01	.12	1.14	.26	
	*F*(9, 91) = 7.41	Corrective: main	−.02	.005	−.42	−4.3	.001	
	*p* < .001	Nonsubstantive: suggestive	.02	.01	.21	1.91	.06	
		Corrective: suggestive	.01	.003	.42	4.1	<.001	.21
	2	Nonsubstantive: main	.02	.01	.22	2.37	.02	
	*F*(8, 87) = 10.16	Corrective: main	−.01	.005	−.22	−2.39	.02	
	*p* < .001	Nonsubstantive: suggestive	.008	.01	.07	.69	.5	
		Corrective: suggestive	.005	.003	.15	1.5	.14	
		Group	.02	.006	.37	3.73	<.001	
		Ground rule: Truth vs. Not	.02	.01	.11	1.26	.21	
		Ground rule: Don’t know	.01	.01	.1	1.06	.29	
		Ground rule: Correct me	.05	.02	.23	2.64	.01	.44
Accuracy during suggestive prompts	1	Nonsubstantive: main	.009	.02	.03	.39	.7	
	*F*(9, 91) = 20.53	Corrective: main	−.03	.01	−.22	−2.74	.007	
	*p* < .001	Nonsubstantive: suggestive	−.06	.03	−.22	−2.3	.02	
		Corrective: suggestive	.05	.007	.59	6.88	<.001	.45
	2	Nonsubstantive: main	.02	.02	.08	1.03	.31	
	*F*(8, 87) = 16.81	Corrective: main	−.006	.01	−.05	−.6	.55	
	*p* < .001	Nonsubstantive: suggestive	−.09	.02	−.33	−3.72	<.001	
		Corrective: suggestive	.03	.007	.4	4.66	<.001	
		Group	.05	.01	.32	3.79	<.001	
		Ground rule: Truth vs. Not	.02	.03	.06	.77	.44	
		Ground rule: Don’t know	.04	.03	.10	1.32	.19	
		Ground rule: Correct me	.04	.04	.07	1.02	.29	.57

## Footnotes

1 Because of interviewer error, two children were not asked the “don’t know” question.
